# Co-existence of fibroadenoma, intraductal carcinoma and invasive ductal carcinoma in the unilateral breast mass: a case report and literature review

**DOI:** 10.3389/fonc.2026.1727815

**Published:** 2026-02-19

**Authors:** Sha Zhong, Hui Xu, Xuan Ye, Yi Xu, Zhe Fan

**Affiliations:** 1 Department of Breast and Thyroid Surgery, Guangzhou Women and Children’s Medical Center, Guangzhou Medical University, Guangzhou, Guangdong, China; 2 Department of Pathology, Guangzhou Women and Children’s Medical Center, Guangzhou Medical University, Guangzhou, Guangdong, China

**Keywords:** breast fibroadenoma, case report, coexistence, ductal carcinoma in situ, invasive ductal carcinoma

## Abstract

**Background:**

Fibroadenoma is the most common benign breast tumor. However, the coexistence of ductal carcinoma *in situ* (DCIS) and invasive ductal carcinoma (IDC) within single breast fibroadenomas is rare.

**Case description:**

A 71-year-old woman presented with a palpable mass in her left breast, and she had a history of right-side breast cancer, which had been treated 16 years ago. Invasive ductal carcinoma was confirmed through core needle biopsy. She subsequently underwent left breast mastectomy with sentinel lymph node biopsy. Postoperative pathological examination revealed two fibroadenomas: the larger one coexisted with IDC and focal DCIS, and the small one coexisted with high-grade DCIS. Immunohistochemical analysis showed positive expression of estrogen receptor (ER) and progesterone receptor (PR), but negative expression of Her-2, demonstrating a Luminal B1 subtype (clinical stage IA). She had completed the adjuvant chemotherapy and currently received endocrine therapy. No recurrence was found during the follow-up examinations.

**Conclusion:**

Coexistence of fibroadenoma, DCIS and IDC in unilateral breast mass is rare, highlighting the importance of comprehensive preoperative evaluation, optimal surgical strategy and accurate histopathological examination.

## 1 Introduction

Breast fibroadenoma is a benign neoplasm characterized by well-circumscribed margins and composed of proliferating stromal and epithelial components (1). It is commonly encountered in clinical practice and can affect women of all age groups, with the highest incidence observed in those aged 20 to 30 years (2). Although fibroadenoma is generally regarded as benign, accumulating evidence indicates an association between this lesion and a modestly increased risk of developing invasive breast cancer (3). Nevertheless, malignant transformation of fibroadenoma into carcinoma remains exceedingly rare. The mean age at diagnosis for carcinoma arising from fibroadenoma ranges from approximately 42 to 44 years, which is about 15 years older than the peak age for simple fibroadenoma (2). Due to the rarity of such occurrences, the clinicopathological features and prognosis of carcinomas that develop within fibroadenomas are not yet fully understood. Herein, we report a previously unreported case in which DCIS, IDC, and fibroadenoma were concurrently identified on histopathological examination. A comprehensive case presentation and a review of the literature are provided.

## 2 Case presentation

In July 2025, a 71-year-old woman presented to our hospital with a palpable mass in the left breast that had been present for two years. Her medical history was significant for right breast cancer diagnosed and treated 16 years earlier. In July 2009, she underwent a right modified radical mastectomy with axillary lymph node dissection for a 15-mm Grade III invasive ductal carcinoma (IDC). Pathological examination of 18 Level I and 8 Level II lymph nodes revealed no evidence of metastasis, and immunohistochemical analysis confirmed a triple-negative phenotype (ER^-^, PR^-^, HER2^-^). Following surgery, she completed six cycles of adjuvant chemotherapy consisting of cyclophosphamide, doxorubicin, and 5-fluorouracil (CTX, ADM, 5-FU) and has since maintained regular follow-up visits.

**Figure 1:**
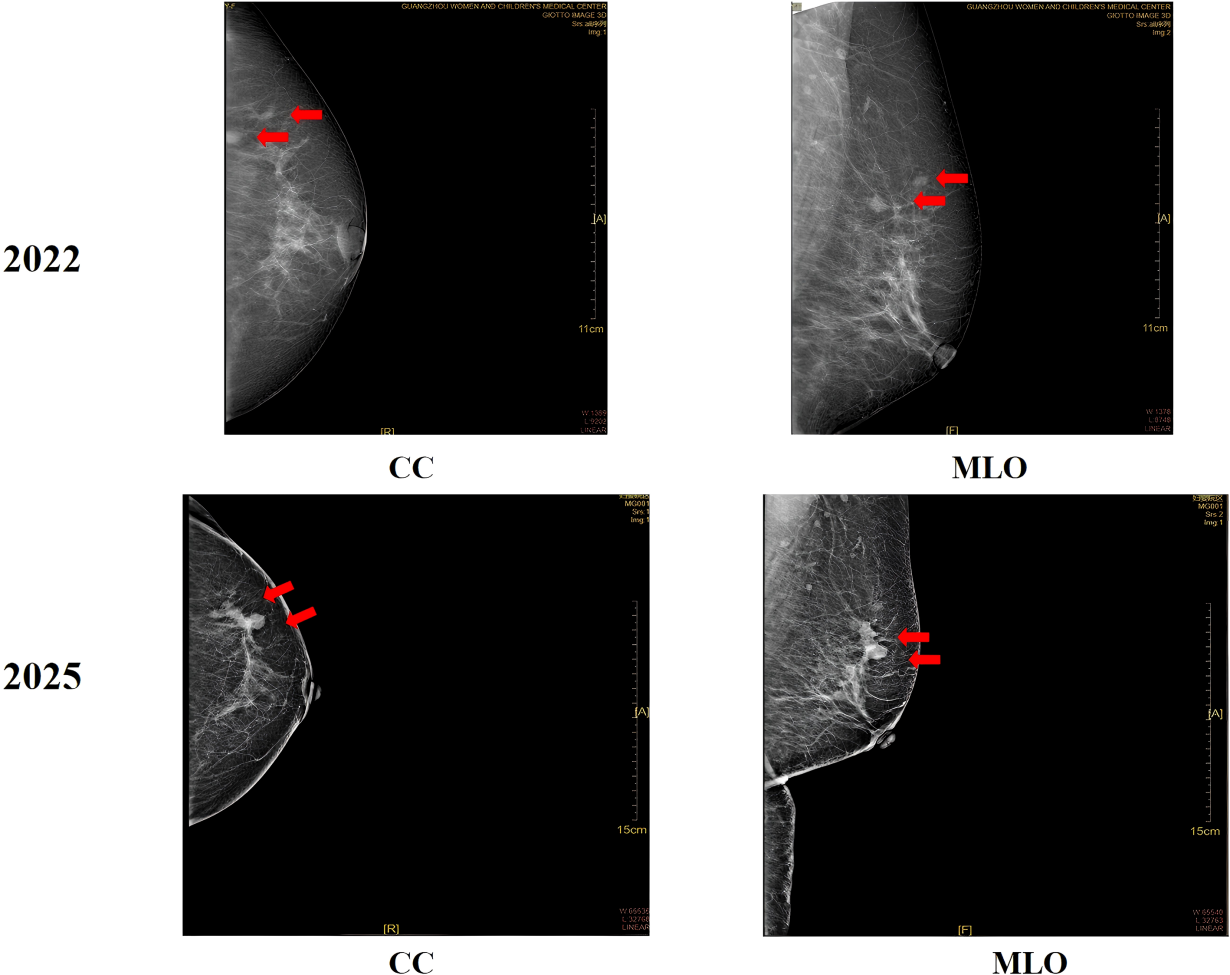
Comparative mammographic views of the left breast in 2022 (upper row) and 2025 (lower row). Upper row, Craniocaudal (CC) and mediolateral oblique (MLO) views from 2022 show two distinct masses (arrows). Lower row, Corresponding CC and MLO views from 2025 demonstrate enlargement and confluence of these masses.

**Figure 2:**
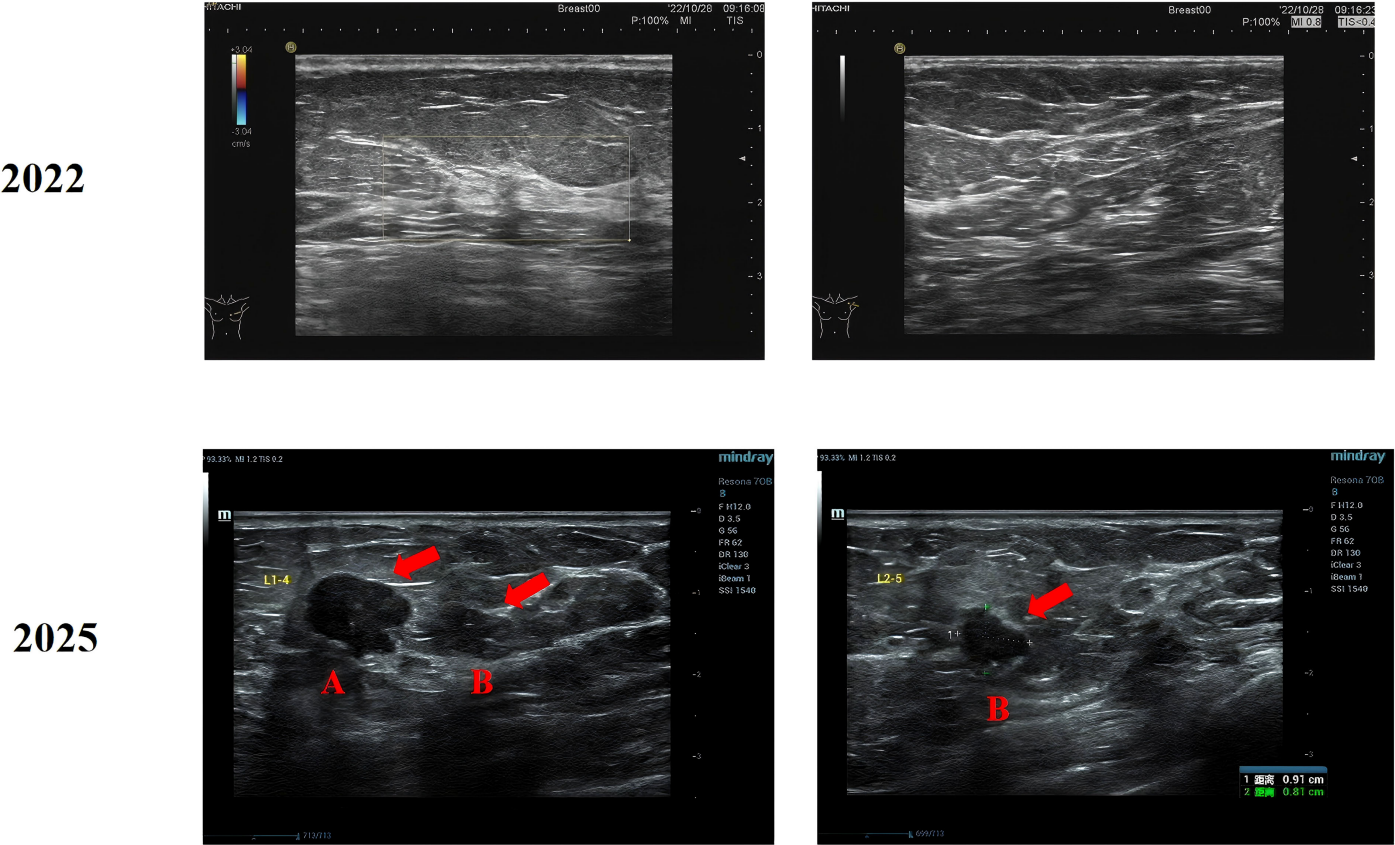
Comparative breast ultrasound images from 2022 and 2025. The 2022 examination revealed no definite space-occupying lesions. In 2025, two distinct masses were observed within the same imaging plane.

In 2022, mammography demonstrated two isolated nodules in the upper outer quadrant of the left breast, measuring 9×8 mm and 8×7 mm, respectively. The nodules exhibited slightly irregular margins without definite suspicious calcifications, and were assessed as BI-RADS 3. Axillary imaging demonstrated several small lymph nodes, the largest measuring 4 mm. The right breast had previously undergone resection and showed no abnormalities on imaging. The patient declined the recommended biopsy and did not attend subsequent follow-up appointments. In February 2025, she noticed a hard, painless mass in the left breast but delayed medical evaluation as the lesion gradually increased in size. Five months later, she presented to our hospital due to progressive enlargement of the mass. Mammography identified three suspicious lesions, measuring 28×16 mm, 9×6 mm, and 8×6 mm, all assigned a BI-RADS 4C classification (**Figure 1**). Breast ultrasound detected two corresponding lesions: one measuring 13×14×10 mm located at the 1 o’clock position, 4 cm from the nipple, and another measuring 9×9×8 mm at the 2 o’clock position, 5 cm from the nipple (**Figure 2**). Both exhibited irregular shapes, ill-defined margins, and scattered strong internal echoes, resulting in a BI-RADS 4A assessment. 

**Figure 3:**
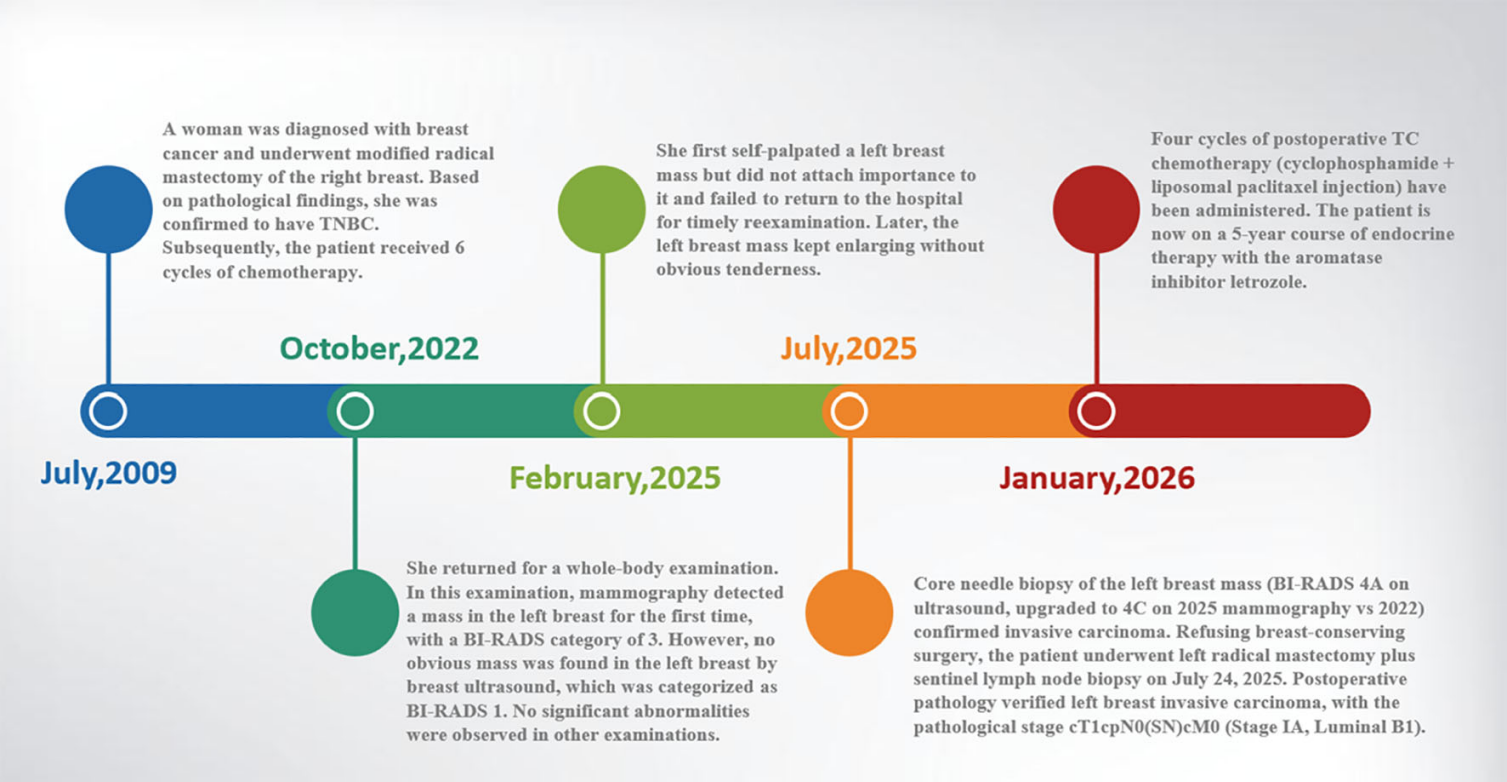
Timeline of events and clinical management process. Additionally, the patient had a long-standing history of hypertension managed with amlodipine besylate and irbesartan. There was no reported family history of malignant tumors.

**Figure 4:**
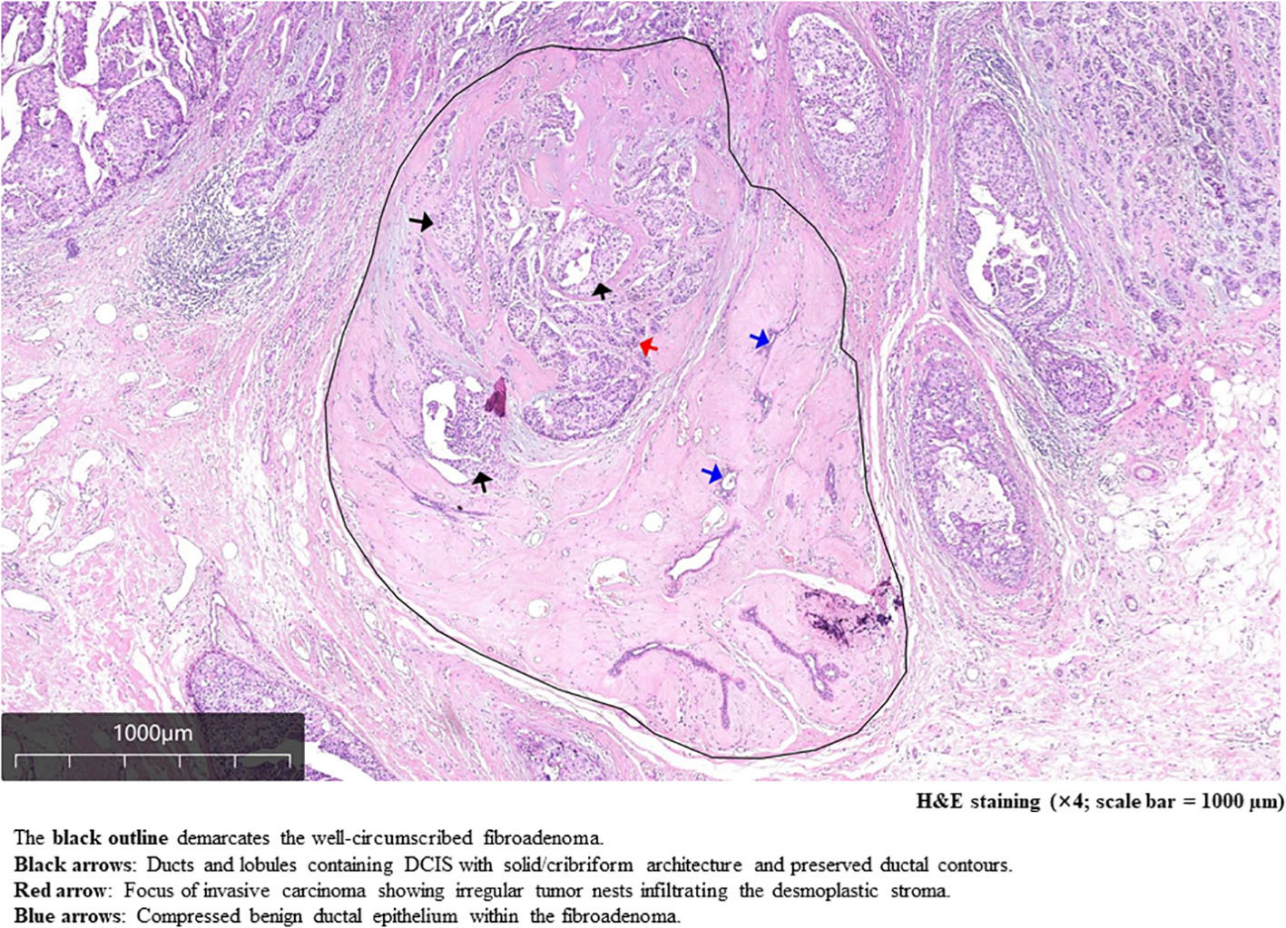
Morphological characteristics and infiltrative pattern of the larger lesion.

**Figure 5:**
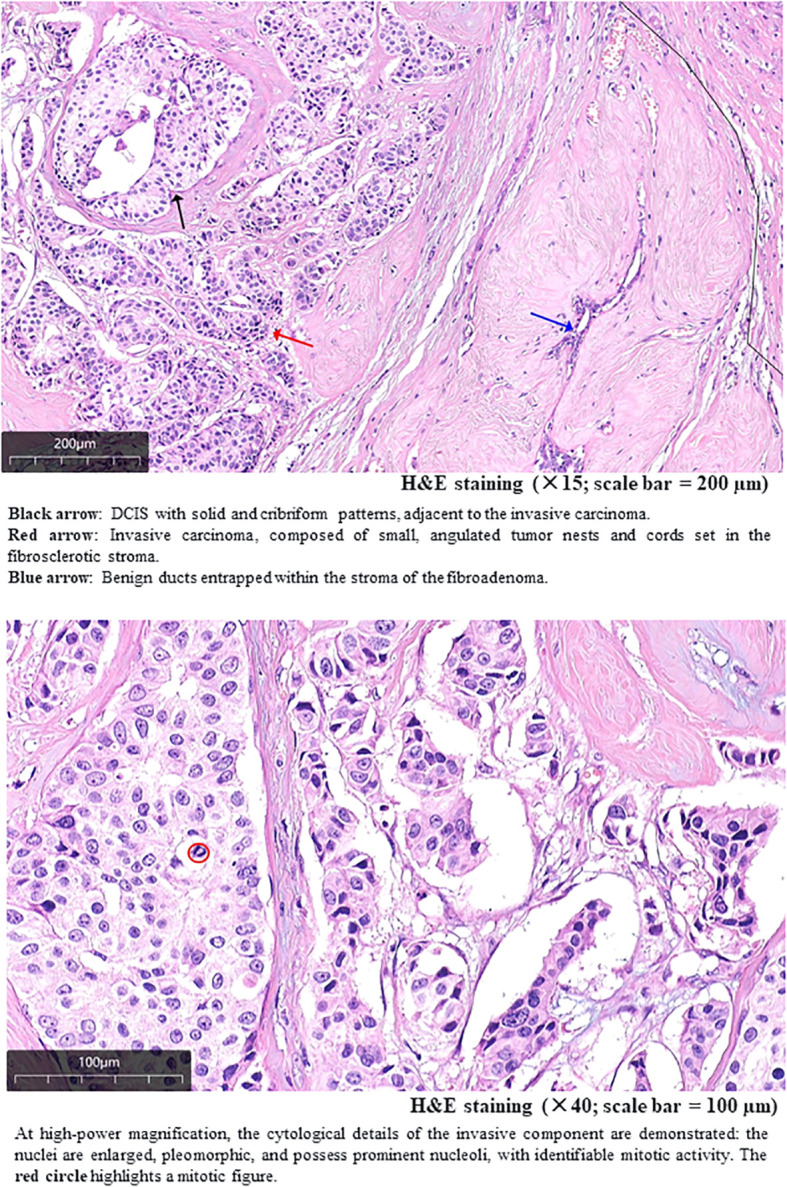
Enlarged view and cytological characteristics of the invasive carcinoma lesion.

**Figure 6:**
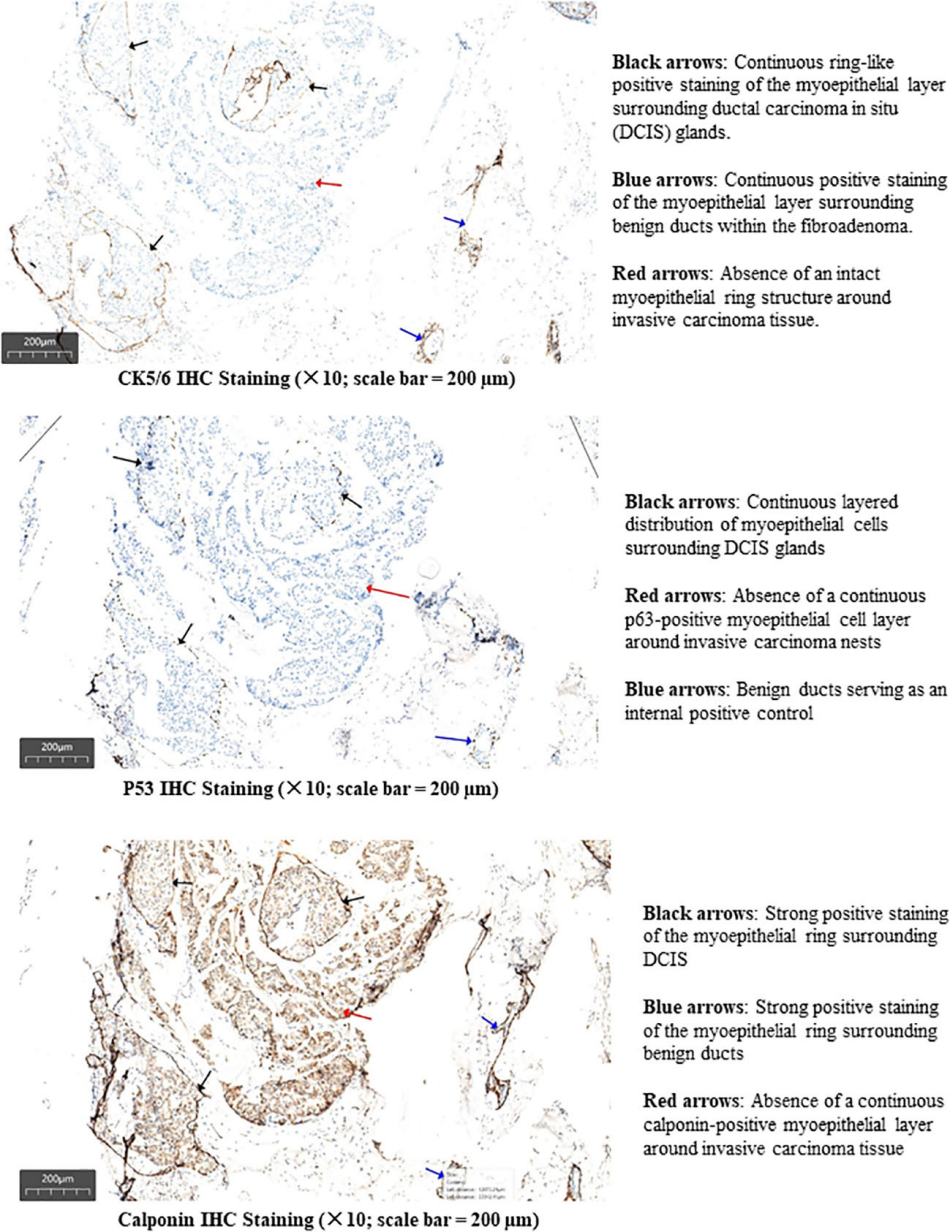
IHC staining for myoepithelial markers.

After a comprehensive evaluation, the patient underwent a Bard Magnum needle biopsy on 22 July 2025, which confirmed a Grade II invasive ductal carcinoma. Given the presence of multifocal lesions and the patient’s preference for non-breast-conserving management, she subsequently underwent a left simple mastectomy with sentinel lymph node biopsy on 24 July 2025. Postoperative pathological examination of the sentinel lymph nodes revealed no evidence of metastasis (**Figure 3**). Final paraffin-embedded section analysis identified two distinct lesions:

1. A 15 × 15 × 10 mm lesion composed of invasive ductal carcinoma, fibroadenoma, and a minor component of DCIS (**Figures 4**, **5**). Immunohistochemical (IHC) analysis demonstrated strong positive expression of estrogen receptor (ER) (+, 90%) and progesterone receptor (PR) (+, 90%), negative human epidermal growth factor receptor 2 (HER2) status (score: 0), and a Ki-67 proliferation index of 50%. No lymphovascular or perineural invasion was observed. Additional IHC staining for myoepithelial markers (CK5/6, p63, and calponin) confirmed the presence of an intact myoepithelial layer around benign ducts and DCIS, and its absence around invasive carcinoma. These findings support the diagnosis of invasive ductal carcinoma in this lesion (**Figure 6**). 2. A breast fibroadenoma associated with high-grade DCIS, measuring approximately 9 × 8 × 7 mm.

Based on the immunohistochemical profile, the tumor was classified as Luminal B1 subtype. According to the American Joint Committee on Cancer (AJCC) staging guideline (4), the tumor-node-metastasis (TNM) classification was determined to be cT1c pN0 cM0, corresponding to Stage IA breast cancer. This malignancy is entirely independent of the contralateral (right-sided) breast cancer diagnosed 16 years earlier and is thus considered a newly diagnosed primary breast cancer, as confirmed by the current histopathological and immunohistochemical findings. , , 

Given the patient’s age and early-stage (Stage IA) disease, we discussed the option of genomic profiling, including Oncotype DX, EndoPredict, or MammaPrint, in line with NCCN guidelines (5). However, the patient declined this testing due to concerns about the cost. Motivated by a strong desire to minimize the risk of recurrence, she opted to proceed with adjuvant chemotherapy.

She subsequently received four cycles of TC chemotherapy (liposomal paclitaxel for injection plus cyclophosphamide) over three months. Upon completion of chemotherapy, adjuvant endocrine therapy was initiated with oral letrozole at a daily dose of 2.5 mg, with a planned duration of five years. A follow-up assessment conducted 6 months postoperatively revealed no evidence of disease recurrence; the patient had completed the chemotherapy regimen and remained on letrozole therapy as per protocol.

## 3 Discussion

Fibroadenoma, a biphasic benign tumor most commonly observed in young women, typically manifests as a palpable mass and often regresses following menopause. It can be subclassified into several histological types, including simple, complex, juvenile, giant, and cellular fibroadenoma. Of these subtypes, complex fibroadenoma is associated with a relatively higher risk of malignant transformation (6). A retrospective study (7) revealed that 31.3% of patients diagnosed with fibroadenoma containing cancerous components were under 35 years of age, leading the researchers to hypothesize that malignant transformation of tissue within fibroadenomas may be associated with high levels of estrogen stimulation in young women. Whether fibroadenoma itself increases the risk of breast cancer remains inconclusive. Current evidence suggests that, compared to women without fibroadenoma, those with fibroadenoma have an approximately 2.17-fold higher risk of developing IDC (8–10). Some studies report that the probability of detecting carcinoma within a fibroadenoma is 0.1% to 0.3% (8, 11), with approximately one-third of these cases being lobular carcinoma, one-third ductal carcinoma or mixed ductal-lobular carcinoma, and the incidences of lobular carcinoma *in situ* (LCIS) and DCIS being roughly equivalent. The risk factors for the malignant transformation of fibroadenoma have long been a focus of research. Hammood et al. (12) have identified that advanced age and a family history of breast cancer are risk factors for carcinogenesis in fibroadenomas. El-Essawy et al. (6) suggest that the development of carcinoma is more closely associated with the degree of epithelial cell proliferation within the fibroadenoma rather than the mere presence of the lesion itself.

In general, it is difficult to distinguish benign fibroadenomas from those undergoing malignant transformation based solely on ultrasound and mammographic findings. However, in cases of fibroadenoma with malignant transformation, certain specific features may be observed on ultrasound examination. Fibroadenomas exhibiting malignant potential may appear on ultrasound as masses with irregular shapes, poorly defined margins, extensive hypoechoic areas, and surrounding echo halos; they may also induce compression or architectural distortion of adjacent tissues. Mammography is more sensitive in detecting lesions containing microcalcifications, which play a crucial role in the early identification of breast fibroadenomas. In contrast, mammography holds a distinct advantage in the early detection of lesions containing microcalcifications, which are often pivotal for identifying fibroadenomas. For instance, in this patient, no distinct mass was identified on breast ultrasound three years ago, whereas mammography at that time revealed a well-demarcated lesion. Studies suggest that the combination of ultrasound and MRI offers the highest diagnostic performance for breast cancer, while mammography and MRI tend to have higher specificity, and ultrasound has higher sensitivity (13). A study by Glechner et al. (14) found that among women with non-dense breasts, there was no difference in the positive detection rate of breast cancer between mammography and breast ultrasound. However, among women with dense breasts, those who additionally underwent breast ultrasound had a higher positive detection rate of breast cancer, but also a higher false-positive rate. Notably, contrast-enhanced MRI has emerged as a potentially effective method for differentiating benign breast fibroadenomas from those with malignant potential (15). Tagliati et al. (16) have further highlighted the utility of the apparent diffusion coefficient (ADC) in distinguishing between benign and malignant fibroadenomas. While simple fibroadenomas typically present with smooth margins and sustained late enhancement on MRI, lesions complicated by carcinoma often show rapid early enhancement and heterogeneous delayed enhancement patterns (17, 18).

In paraffin-embedded pathological diagnoses, fibroadenomas associated with DCIS or IDC are typically incidental findings identified following surgical resection and remain relatively uncommon. Currently, there are no established clinical guidelines specifically addressing this condition. Notably, when fibroadenomas coexist with DCIS or malignant components, the three pathological elements are often intermingled within the same paraffin section, posing significant challenges in accurately measuring the diameter of the invasive carcinoma. As a result, clinical staging may vary according to the assessed tumor size. In such cases, treatment strategies should be guided by the histopathological features of the carcinoma. Specific therapeutic approaches, including surgery, radiotherapy, chemotherapy, and endocrine therapy, are determined based on the clinical stage of the carcinoma and the presence or absence of metastatic disease.

The development and prognosis of fibroadenomas coexisting with DCIS or IDC are infrequently reported in the literature. Clinically, even when imaging findings on ultrasound or mammography suggest benign characteristics, a high index of suspicion should be maintained, and patients should undergo regular follow-up evaluations. If postoperative pathological examination reveals malignant components, the treatment strategy will differ substantially from that of typical fibroadenomas, and the clinical prognosis will also vary significantly.

The uniqueness of this case lies in the coexistence of three distinct pathological entities—fibroadenoma, DCIS, and IDC—within the same set of postoperative pathological specimens, encompassing multiple histological slides from the patient. Immunohistochemical analysis enables the differentiation of these various pathological components within the tumor mass; however, it does not definitively establish whether the invasive ductal carcinoma arises from the epithelial elements within the fibroadenoma. Nevertheless, by integrating findings from the current paraffin-embedded sections with those reported in previous cases, it is clearly evident that the fibroadenoma harbors both DCIS and invasive carcinoma components.

The origin of cancer cells within fibroadenomas has long been a subject of considerable interest among experts; however, to date, no definitive conclusion has been established. Theoretically, two potential etiologies may account for the development of malignant tissue within fibroadenomas. First, breast cancer may originate from the epithelial components of the fibroadenoma, wherein epithelial cells transform into DCIS, which may subsequently progress to invasive carcinoma. Second, the malignant elements and the fibroadenoma may coexist independently, with the carcinoma developing either within or adjacent to the fibroadenoma purely by chance. Xu et al. (7) reported that 62.5% of cases involving malignant transformation in fibroadenomas are associated with carcinoma *in situ*, suggesting that the carcinomatous components primarily arise within the fibroadenoma itself. Academic research is grounded in objective evidence; therefore, it is not possible to definitively conclude that invasive carcinoma originates through a stepwise progression from fibroadenoma to carcinoma *in situ* and subsequently to invasive carcinoma, based solely on the co-occurrence of these three components in pathological specimens. Current technological limitations preclude such conclusive determination. Notably, an increasing number of experts and scholars have begun exploring the origins of breast cancer at a more microscopic and molecular level. Chen et al. (19) indicate that promoter hypermethylation represents an early event in breast carcinogenesis, and the methylation of RASSF1A and APC occurs more frequently in tumor samples from women with benign breast diseases who are at high risk of malignant transformation compared to those at moderate or low risk.

Regarding the clinical prognosis of this condition, it is observed that detailed descriptions of prognostic outcomes are lacking in the majority of reported cases. Nevertheless, there is a broad consensus among experts that the clinical course primarily hinges on the patient’s clinical stage, molecular subtype, and the extent to which treatment adheres to standardized protocols. Management should be guided by established guidelines for DCIS or IDC, encompassing modalities such as surgery, radiotherapy, systemic therapy, and endocrine therapy, individualized according to the pathological characteristics, molecular classification, and clinical stage (5).

Our study has several limitations. First, as a retrospective analysis of a single, rare case, the generalizability of the findings is inherently constrained. Second, the absence of preoperative breast MRI may have limited the comprehensive assessment of this complex co-pathology. Lastly, the current follow-up period is relatively brief, lasting approximately 6 months; therefore, extended long-term surveillance is essential to fully understand the long-term prognosis of this rare entity. In conclusion, fibroadenomas harboring carcinomatous components represent a rare manifestation of breast pathology, typically identified incidentally through histopathological examination. The uniqueness of this case report lies in the definitive histopathological demonstration of the coexistence of fibroadenoma, DCIS, and IDC. To date, the precise origin of malignant elements within fibroadenomas remains unclear. With continued advancements in pathological diagnostic techniques, it is anticipated that future research will yield improved methods for detecting and elucidating the underlying mechanisms of this uncommon phenomenon.

## Data Availability

The datasets presented in this study can be found in online repositories. The names of the repository/repositories and accession number(s) can be found in the article/supplementary material.
